# Supplementary figures and images for: Phosphorylated viral protein evades plant immunity through interfering the function of RNA-binding protein
Source: PLoS Pathog. 2022 Mar 16;18(3):e1010412. doi: 10.1371/journal.ppat.1010412 (PMC8959173; doi:10.1371/journal.ppat.1010412)

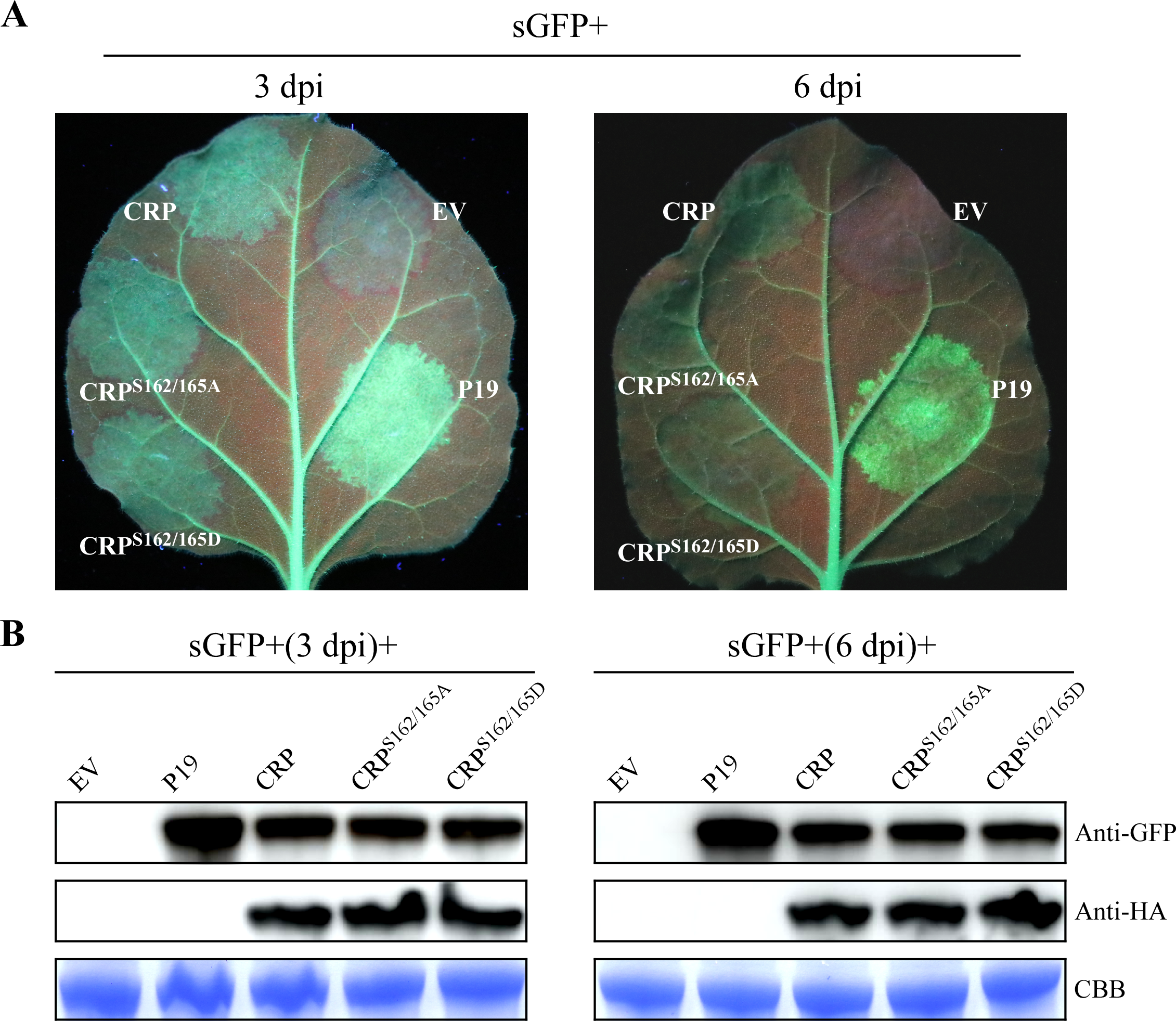

Supplement: S1 Fig — A. sGFP was co-expressed with CRP, CRPS162/165A (a non-phosphorylatable CRP), CRPS162/165D (a phosphorylatable CRP), P19, or an empty vector (EV) in the leaves of 16c transgenic N. benthamiana plants. The infiltrated leaves were photographed at 3 (left) and 6 dpi (right) under a long-wave UV light. B. Detection of GFP in the infiltrated leaf tissues through western blot analysis at 3 (left) and 6 dpi (right) using an anti-GFP or an anti-HA antibody. The CBB-stained gel is used to show sample loadings. (TIF) [file ppat.1010412.s001.tif]

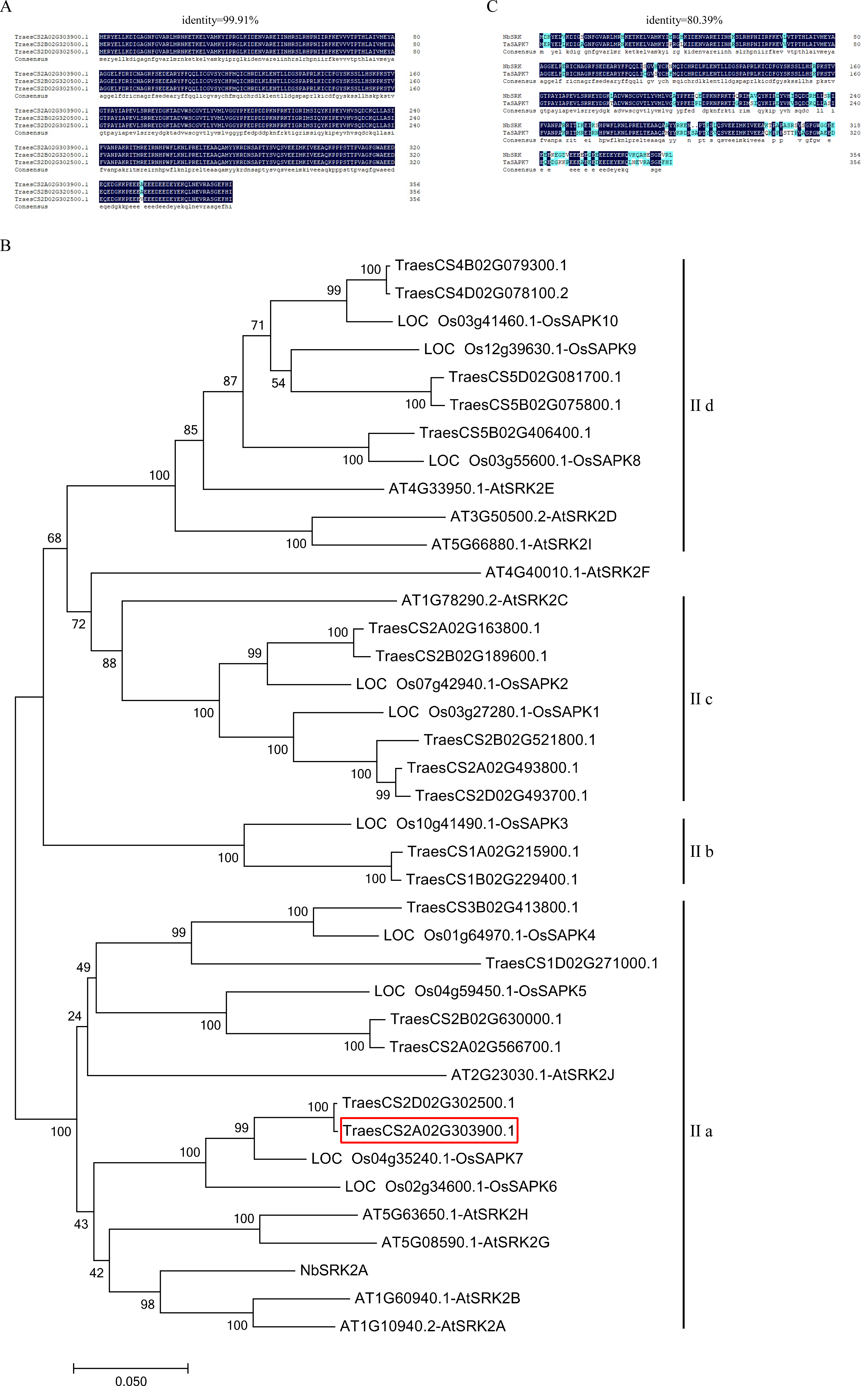

Supplement: S2 Fig — A. Alignment using three wheat SAPK7 sequences. The conserved amino acid (aa) residues are shown in black, while the variable aa residues are shown in blue. B. Maximum likelihood tree of SnRK2 proteins based on amino acid sequences of kinase domain. Bootstrap values were estimated based on 1,000 replications. C. Alignment using NbSRK and TaSAPK7 (TraesCS2A02G303900.1) sequences. (TIF) [file ppat.1010412.s002.tif]

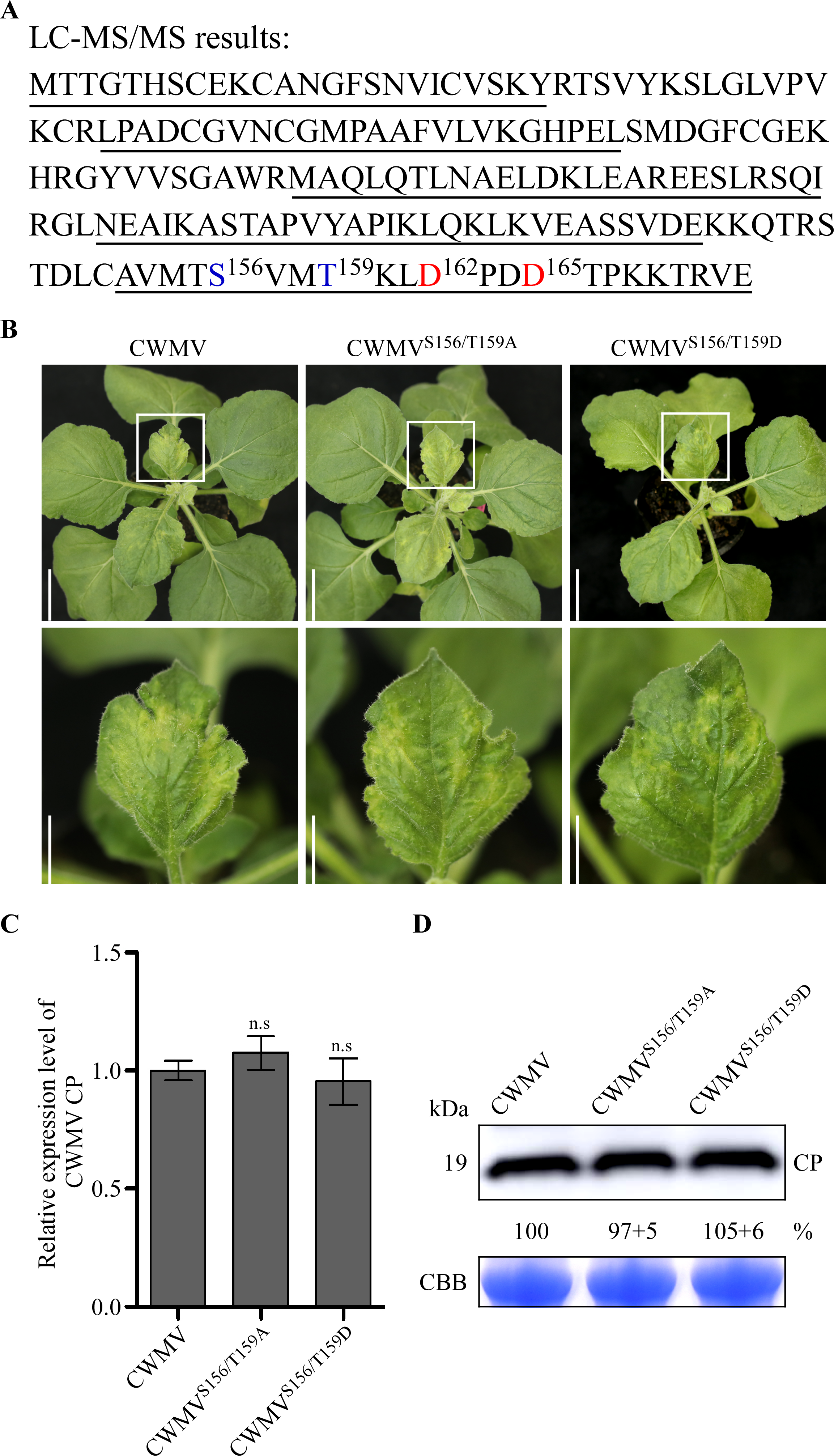

Supplement: S3 Fig — A. LC-MS/MS analysis of the phosphorylation status of CRPS162/165D mutant. The underlined CRPS162/165D amino acid sequence was identified in this study through LC-MS/MS, and the secondary phosphorylation sites in this protein are shown in blue. B. Systemic mosaic symptoms in the CWMV-, CWMVS156/T159A- or CWMVS156/T159D-infected N. benthamiana plants. Photographs were taken at 21 dpi. Scale bar = 5 cm (upper panel), Scale bar = 2 cm (lower panel). C. Relative expression level of CWMV CP in the assayed N. benthamiana plants, determined through qRT-PCR using CWMV CP gene specific primers. The data presented are the means ± standard deviations (SD), calculated using the Student’s t-test. Each treatment had three biological replicates. n.s, no significant difference. D. Accumulation of CWMV CP in the assayed N. benthamiana leaf samples was determined through western blot analysis using a CWMV CP specific antibody. The CBB-stained gel is used to show sample loadings. (TIF) [file ppat.1010412.s003.tif]

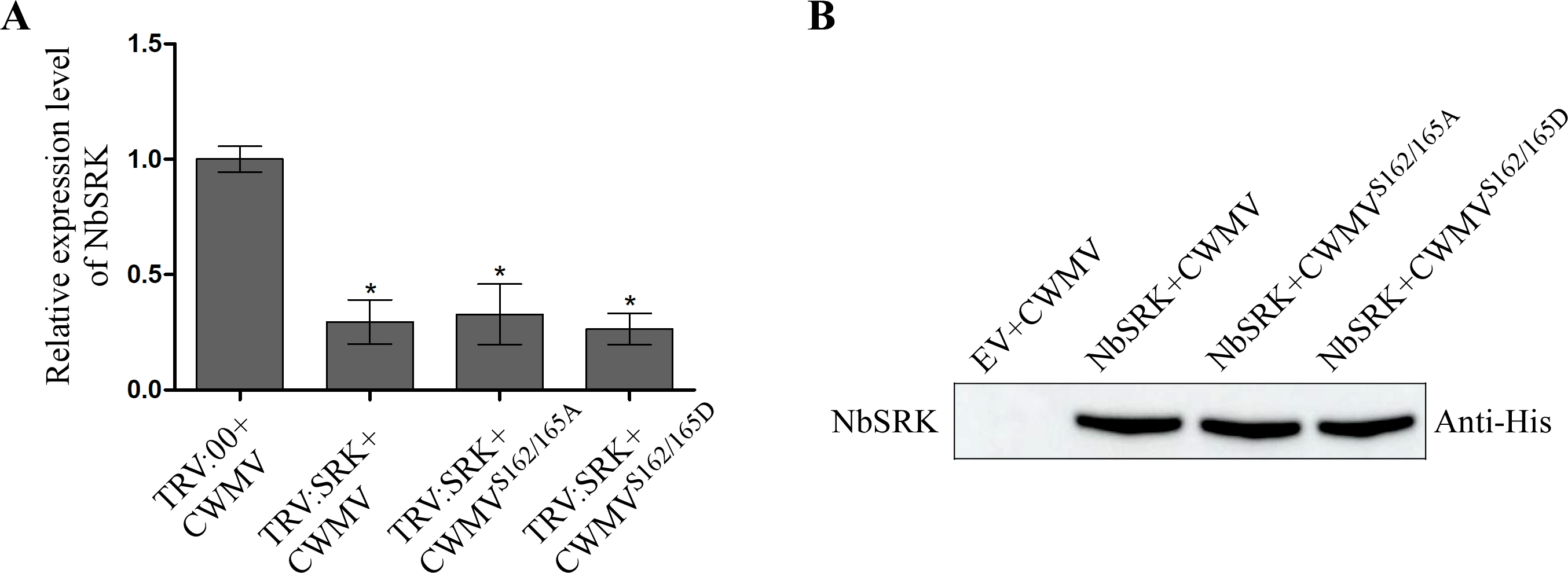

Supplement: S4 Fig — A. Relative expression of NbSRK mRNA in the TRV:00+CWMV-, TRV:NbSRK+CWMV-, TRV:NbSRK+CWMVS162/165A- or TRV:NbSRK+CWMVS162/165D-inoculated N. benthamiana plants was determined through qRT-PCR. The data presented are the means ± SD, determined using the Student’s t-test. Each treatment had three biological replicates. *, P <0.05. B. Detection of NbSRK protein accumulation in the N. benthamiana leaves co-expressing EV+CWMV, NbSRK+CWMV, NbSRK+CWMVS162/165A or NbSRK+CWMVS162/165D through western blot analysis using an anti-His antibody. (TIF) [file ppat.1010412.s004.tif]

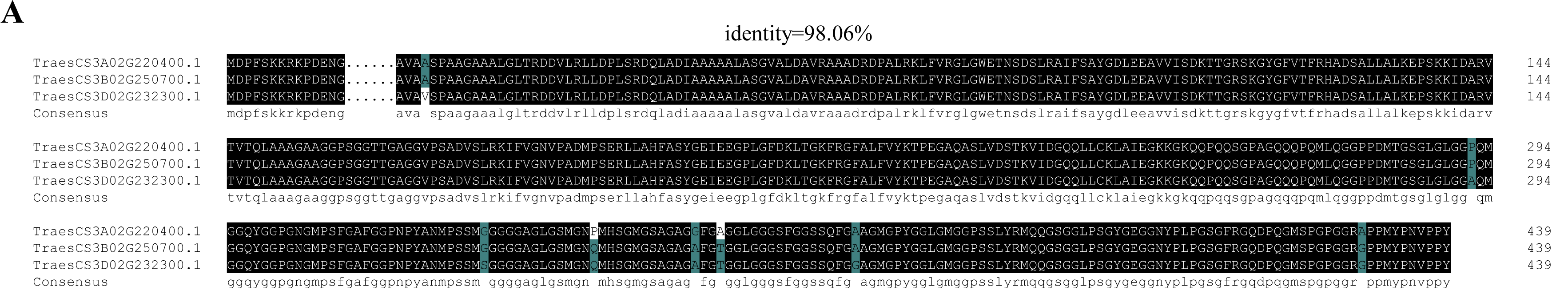

Supplement: S5 Fig — A. Alignment using three TaUBA2C sequences. The conserved aa residues are shown in black, while the variable aa residues are shown in blue. (TIF) [file ppat.1010412.s005.tif]

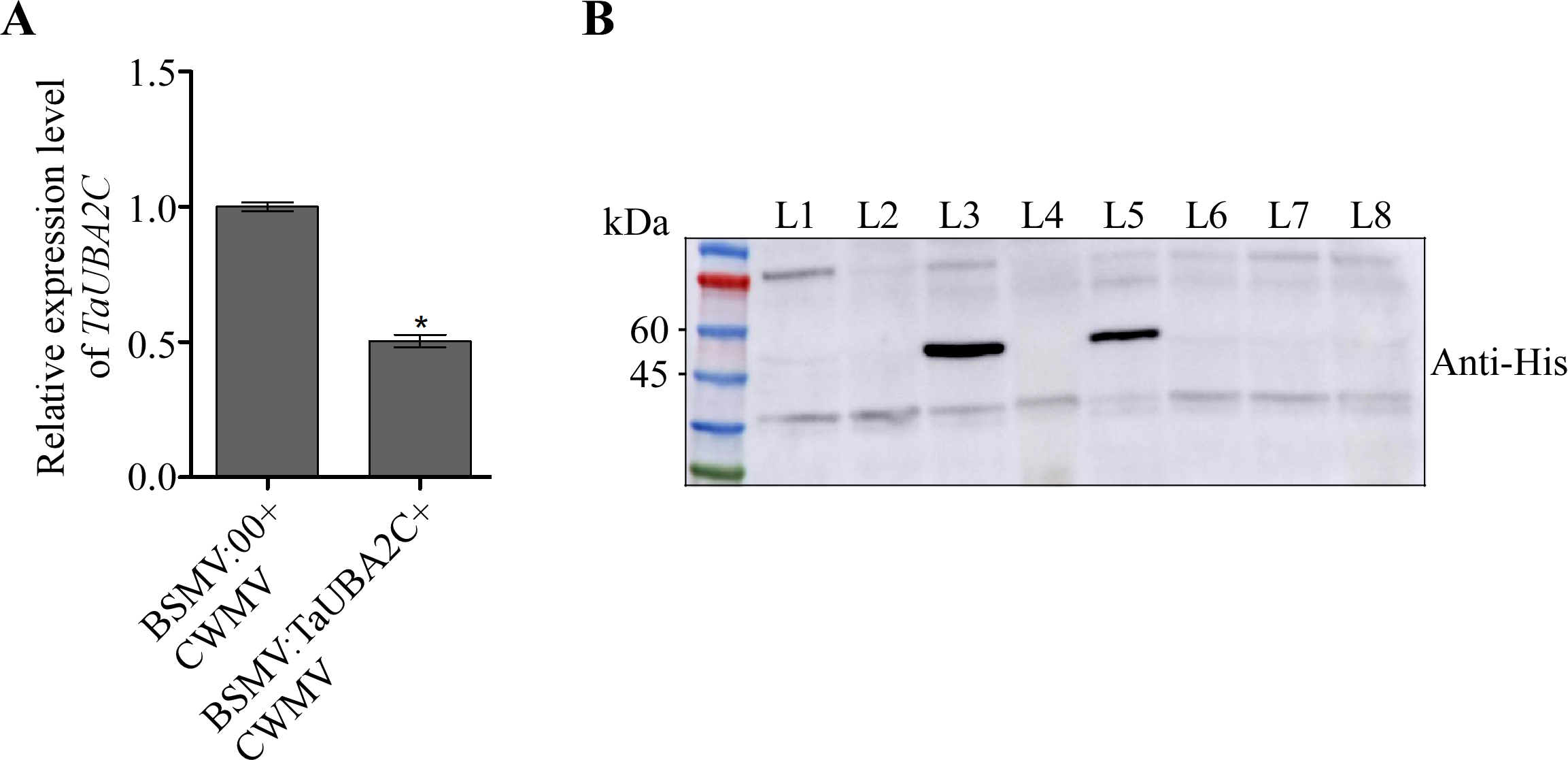

Supplement: S6 Fig — A. Relative expression levels of TaUBA2C mRNA in the BSMV:00+CWMV- or BSMV:TaUBA2C+CWMV-inoculated wheat plants were determined through qRT-PCR at 10 days post BSMV inoculation. The data presented are the means ± SD, determined using the Student’s t-test. Each treatment had three biological replicates. *, P <0.05. B. The expression levels of TaUBA2C in eight transgenic wheat lines were determined through western blot analysis using an anti-His antibody. (TIF) [file ppat.1010412.s006.tif]

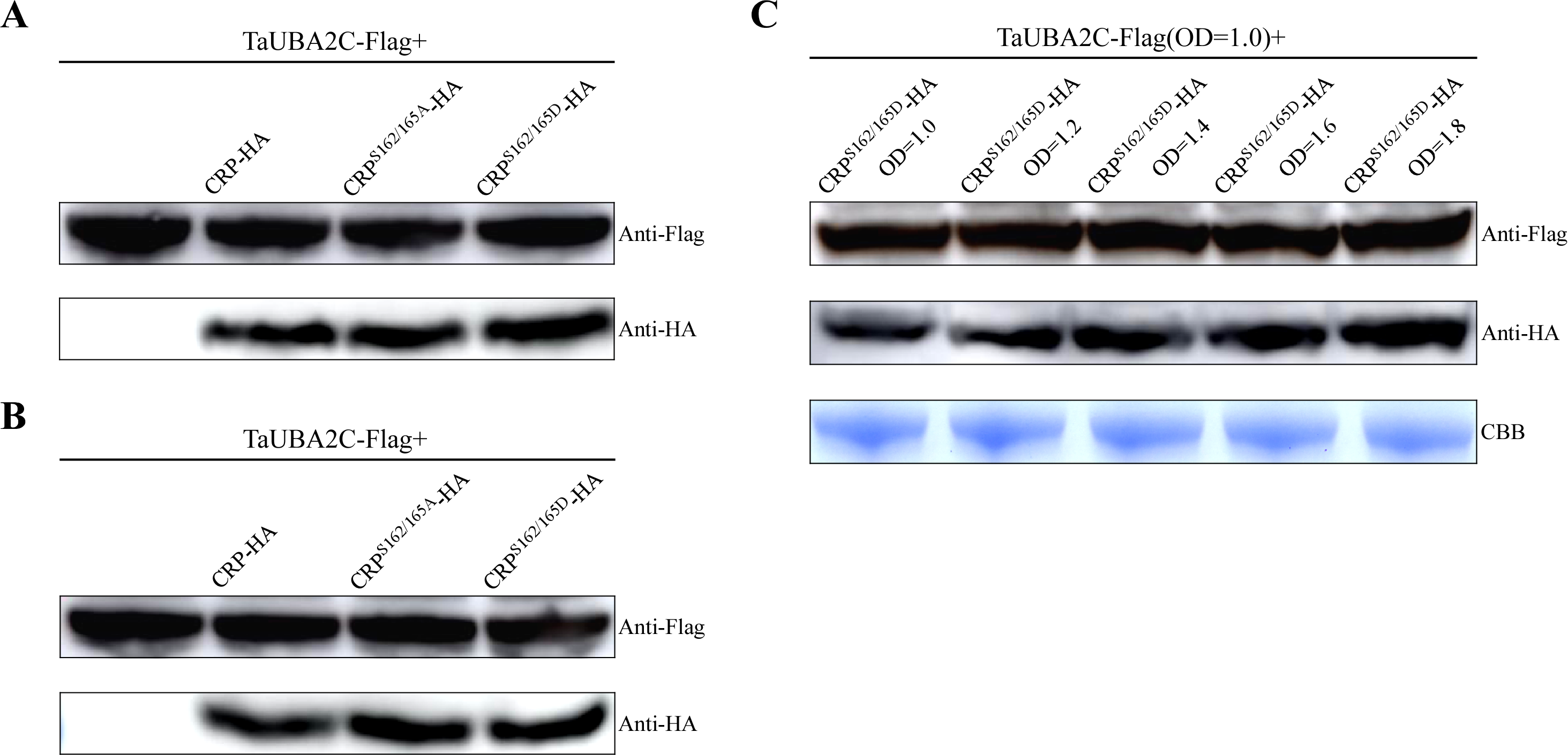

Supplement: S7 Fig — A and B. Western blot analyses of TaUBA2C, CRP, CRPS162/165A and CRPS162/165D expressions in plants using an anti-Flag or an anti-HA antibody. C. Western blot analysis of TaUBA2C and CRPS162/165D expressions in N. benthamiana leaves co-inoculated with TaUBA2C and different concentrations of CRPS162/165D using an anti-Flag or an anti-HA antibody. The CBB-stained gel is used to show sample loadings. (TIF) [file ppat.1010412.s007.tif]

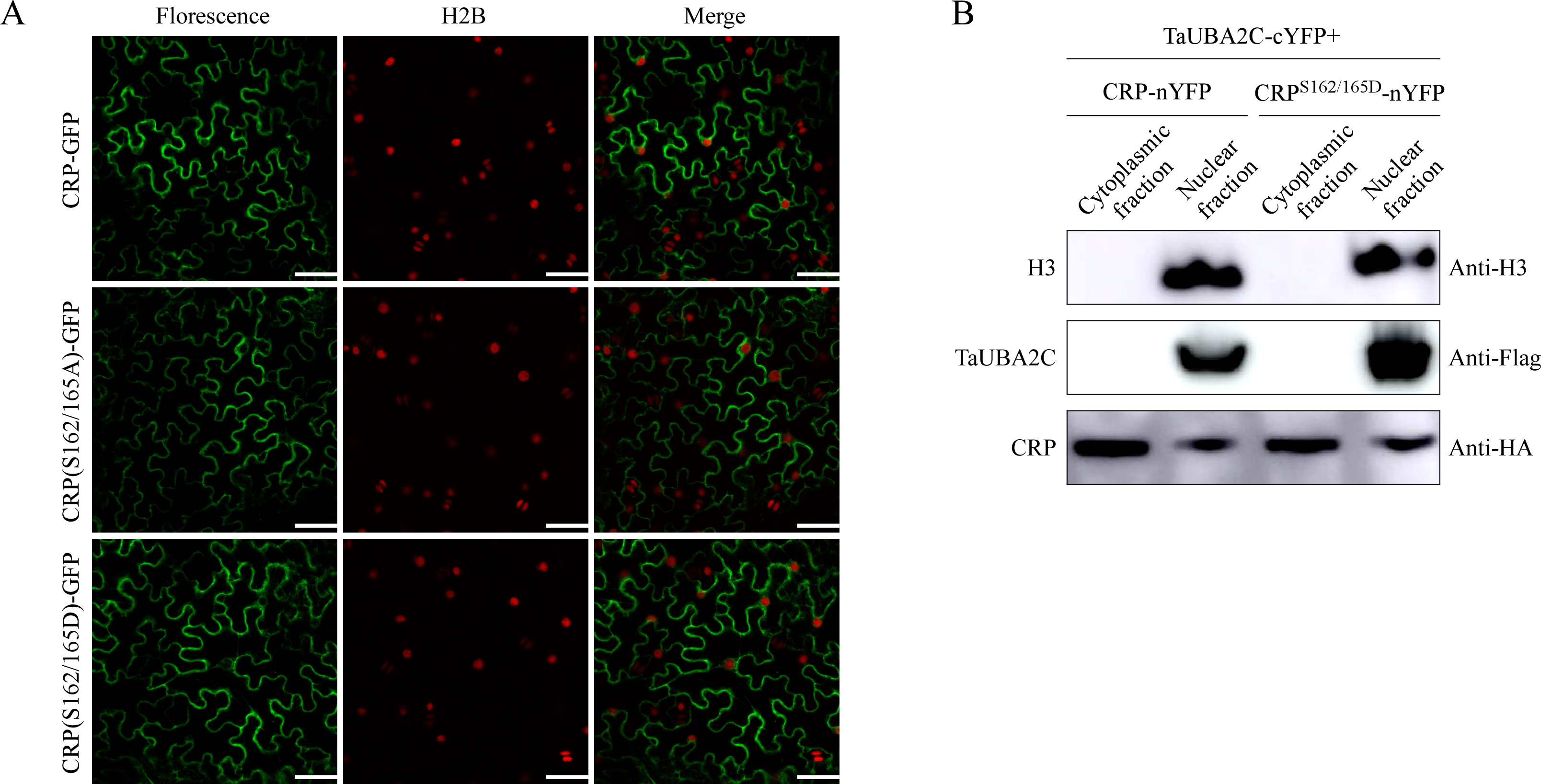

Supplement: S8 Fig — A. Subcellular localization patterns of CRP-GFP, CRPS162/165A-GFP and CRPS162/165D-GFP in N. benthamiana leaf epidermal cells. These proteins were expressed individually in the leaves of the H2B-RFP transgenic N. benthamiana plants. Confocal images were taken at 60 hpi. Scale bar = 50 μm. B. Western blot analyses of subcellular localizations of the co-expressed TaUBA2C-cYFP and CRP-nYFP, TaUBA2C-cYFP and CRPS162/165D-nYFP using an anti-Flag or an anti-HA antibody. Histone H3 protein was used as a nuclear protein marker. (TIF) [file ppat.1010412.s008.tif]

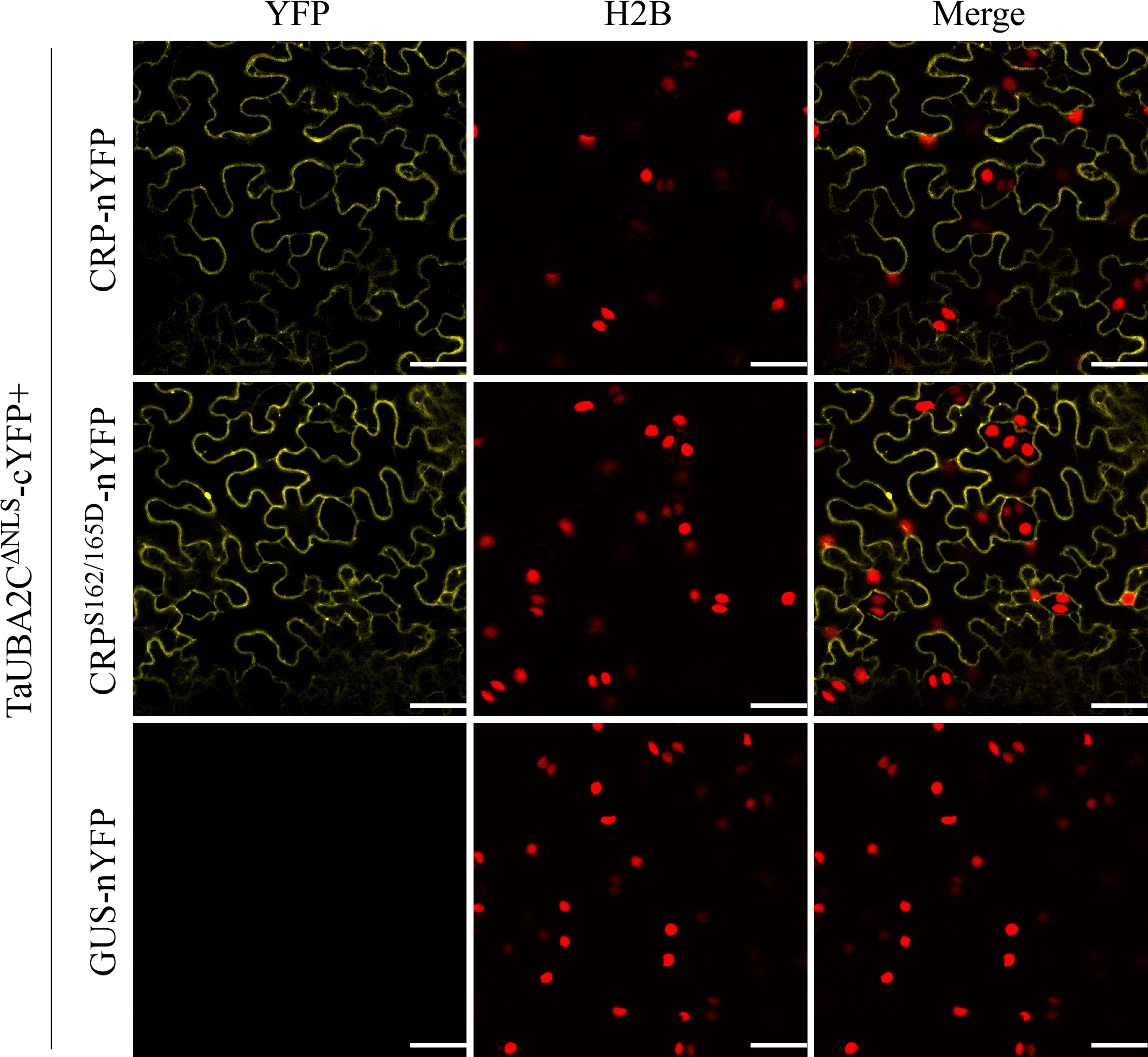

Supplement: S9 Fig — BiFC analysis with TaUBA2CΔNLS-cYFP and CRP-nYFP, CRPS162/165D-nYFP or GUS-cYFP in H2B-RFP transgenic N. benthamiana leaves. TaUBA2CΔNLS: nuclear localization signal deletion mutant of TaUBA2C. Confocal images were taken at 60 hpi. Scale bar = 50 μm. (TIF) [file ppat.1010412.s009.tif]

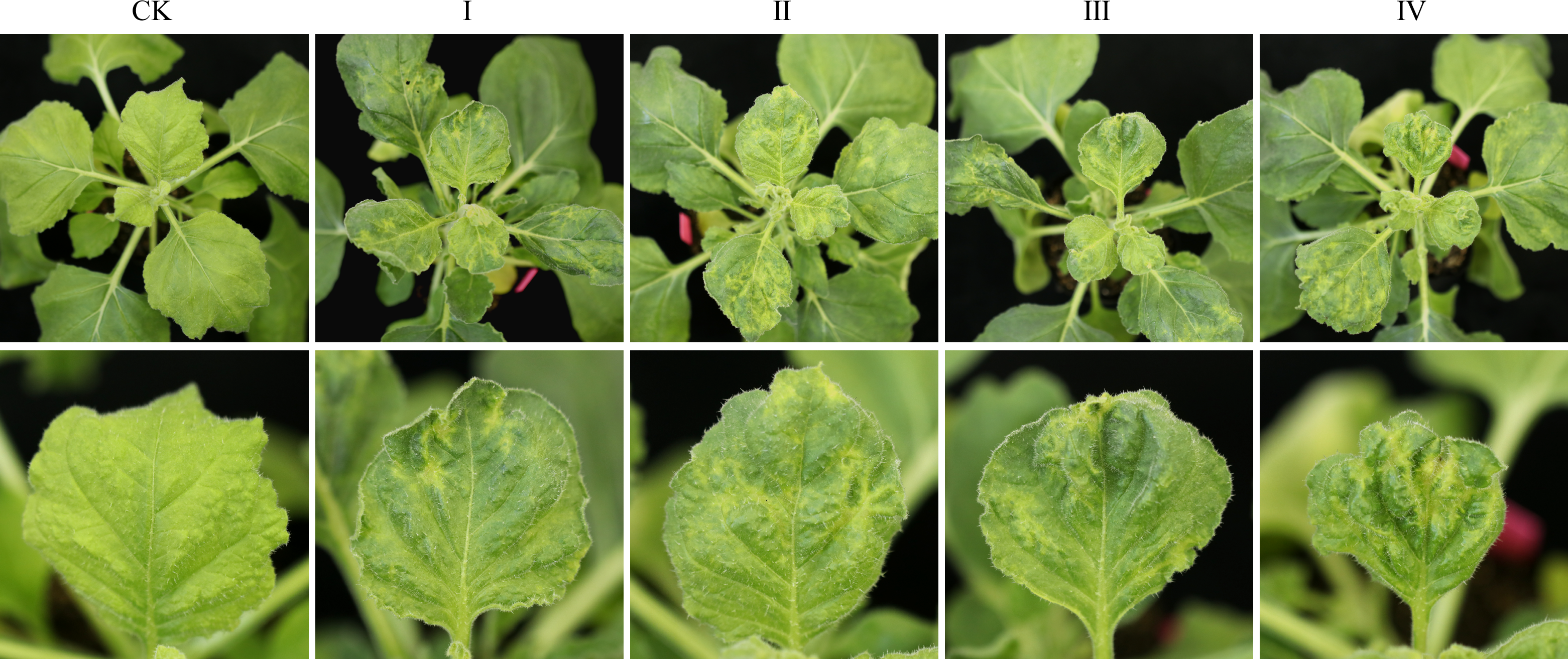

Supplement: S10 Fig — (TIF) [file ppat.1010412.s010.tif]
