# Supplementary material for: Phosphorylated viral protein evades plant immunity through interfering the function of RNA-binding protein
Source: PLoS Pathog. 2022 Mar 16;18(3):e1010412. doi: 10.1371/journal.ppat.1010412 (PMC8959173; doi:10.1371/journal.ppat.1010412)
Supplement: S1 Table — (DOCX) [file ppat.1010412.s011.docx]

Supplemental Table 1. The complete phospho-peptide sequences and mass-to-charge ratio (m/z) from the mass spectrum assay. Related to Figure 1B

| Peptide sequense | Peptide modified sequence | m/z | z |
| --- | --- | --- | --- |
| S16 | CANGFS(+79.97)NVICVSK | 576.2968 | 2 |
| S65 | KGHPELS(+79.97)MDGFCGEKH | 830.3051 | 2 |
|  | KGHPELS(+79.97)MDGFCGEKH | 822.3161 | 2 |
| S80 | RGYVVS(+79.97)GAWRMAQLQTLNAELDK | 796.0712 | 3 |
| T90 | T(+79.97)LNAELDKLVAR | 803.3381 | 2 |
| S107 | RS(+79.97)QIRGLNEAIKASTAP | 817.0285 | 3 |
| T145 | LKVEAFNVDEKIQT(+79.97)R | 816.6989 | 3 |
| S162 | STDLCAVMTSVMTKLS(+79.97)PDSTPKK | 803.3398 | 2 |
|  | RSTDLCAVMTSVMTKLS(+79.97)PDSTPKK | 873.733 | 3 |
|  | RSTDLCAVMTSVMTKLS(+79.97)PDSTPKK | 822.3682 | 3 |
|  | RSTDLCAVMTSVMTKLS(+79.97)PDSTPKKT | 879.0643 | 3 |
|  | RSTDLCAVMTSVMTKLS(+79.97)PDSTPKK | 822.3659 | 3 |
|  | RSTDLCAVMTSVMTKLS(+79.97)PDSTPKK | 817.0359 | 3 |
| S165 | RSTDLCAVMTSVMTKLSPDS(+79.97)TPKK | 822.3682 | 3 |
|  | RSTDLCAVMTSVMTKLSPDS(+79.97)TPKKT | 879.0643 | 3 |
|  | RSTDLCAVMTSVMTKLSPDS(+79.97)TPKK | 822.3659 | 3 |
|  | RSTDLCAVMTSVMTKLSPDS(+79.97)TPKK | 817.0359 | 3 |
|  | RSTDLCAVMTSVMTKLSPDS(+79.97)TPKK | 849.7217 | 3 |
|  | RSTDLCAVMTSVMTKLSPDS(+79.97)TPKK | 835.7061 | 3 |
