# Supplementary material for: Phosphorylated viral protein evades plant immunity through interfering the function of RNA-binding protein
Source: PLoS Pathog. 2022 Mar 16;18(3):e1010412. doi: 10.1371/journal.ppat.1010412 (PMC8959173; doi:10.1371/journal.ppat.1010412)
Supplement: S2 Table — (DOCX) [file ppat.1010412.s012.docx]

| Number | Accession | Description | Number of clones |
| --- | --- | --- | --- |
| 1 | TraesCS2A02G303900.1 | serine/threonine-protein kinase SAPK7 | 6 |
| 2 | TraesCS3A02G220400.1 | UBP1-associated protein 2C | 3 |
| 3 | TraesCS5D02G093900 | heat shock cognate 70 kDa protein 2-like | 3 |
| 4 | TraesCS3D02G220900.1 | elongation factor 2-like | 2 |
| 5 | TraesCS5B02G258900.2 | HSP80-2 | 1 |

Supplemental Table 2. The total of 15 positive clones obtained from the Y2H screening with CRP as bait.
