# Supplementary material for: Phosphorylated viral protein evades plant immunity through interfering the function of RNA-binding protein
Source: PLoS Pathog. 2022 Mar 16;18(3):e1010412. doi: 10.1371/journal.ppat.1010412 (PMC8959173; doi:10.1371/journal.ppat.1010412)
Supplement: S3 Table — (DOCX) [file ppat.1010412.s013.docx]

S3 Table Primers used in this study

| **Name** | **Primers (5'-3')** | **Description** |
| --- | --- | --- |
| CWMV-R2F2910 | CAACTTCGCACTTTAAATGCTGAG | forward primer for CWMV^S162A^ by overlap-PCR |
| CWMV-R2R3155 | CTTCGGTGAGGAATCAGTTGCCAGCTTAGC | reverse primer for CWMV^S162A^ by overlap-PCR |
| CWMV-R2F3135 | GCAACTGATTCCTCACCGAAG | forward primer for CWMV^S162A^ by overlap-PCR |
| CWMV-R2RBamHI | CGGGATCCTGGGCCGGTTTACCCACC | reverse primer for CWMV^S162A^ by overlap-PCR |
| CWMV-R2F2910 | CAACTTCGCACTTTAAATGCTGAG | forward primer for CWMV^S165A^ by overlap-PCR |
| CWMV-R2R3165 | CACGAGTCTTCTTCGGTGAGGCATCAGTTGAC | reverse primer for CWMV^S165A^ by overlap-PCR |
| CWMV-R2F3134 | GCCTCACCGAAGAAGACTCGTG | forward primer for CWMV^S165A^ by overlap-PCR |
| CWMV-R2RBamHI | CGGGATCCTGGGCCGGTTTACCCACC | reverse primer for CWMV^S165A^ by overlap-PCR |
| CWMV-R2F2910 | CAACTTCGCACTTTAAATGCTGAG | forward primer for CWMV^S162D^ by overlap-PCR |
| CWMV-R2R3155 | CTTCGGTGAGGAATCAGTTGcCAGCTTAGC | reverse primer for CWMV^S162D^ by overlap-PCR |
| DCWMV-R2F3135 | GATACTGATTCCTCACCGAAG | forward primer for CWMV^S162D^ by overlap-PCR |
| CWMV-R2RBamHI | CGGGATCCTGGGCCGGTTTACCCACC | reverse primer for CWMV^S162D^ by overlap-PCR |
| CWMV-R2F2910 | CAACTTCGCACTTTAAATGCTGAG | forward primer for CWMV^S165D^ by overlap-PCR |
| CWMV-R2R3165 | CACGAGTCTTCTTCGGTGAGGCATCAGTTGAC | reverse primer for CWMV^S165D^ by overlap-PCR |
| DCWMV-R2F3134 | GATTCACCGAAGAAGACTCGTG | forward primer for CWMV^S165D^ by overlap-PCR |
| CWMV-R2RBamHI | CGGGATCCTGGGCCGGTTTACCCACC | reverse primer for CWMV^S165D^ by overlap-PCR |
| CWMV-R2F2910 | CAACTTCGCACTTTAAATGCTGAG | forward primer for CWMV^S162/165D^ by overlap-PCR |
| CWMV-R2R3155 | CTTCGGTGAGGAATCAGTTGCCAGCTTAGC | reverse primer for CWMV^S162/165D^ by overlap-PCR |
| DDCWMV-R2F3135 | GATACTGATGATTCACCGAAGAAGACT | forward primer for CWMV^S162/165D^ by overlap-PCR |
| CWMV-R2RBamHI | CGGGATCCTGGGCCGGTTTACCCACC | reverse primer for CWMV^S162/165D^ by overlap-PCR |
| attB1C19K | GGGGACAAGTTTGTACAAAAAAGCAGGCTGCATGACTACTGGTACTCATTCT | forward primer for Donr-CRP by gateway |
| attB2C19K | GGGGACCACTTTGTACAAGAAAGCTGGGTCCTCCACACGAGTTTTTTTCGG | reverse primer for Donr-CRP by gateway |
| pET32a-CRP-F | ACAAGGCCATGGCTGATATCGGATCCATGACTACTG GTACTCATTCT | forward primer for pET32a-CRP |
| pET32a-CRP-R | TCGAGTGCGGCCGCAAGCTTGTCGACGCTCCACACGAGTCTTCTTCGG | reverse primer for pET32a-CRP |
| BD-CRP-F | TCAGAGGAGGACCTGCATATGATGACTACTG GTACTCATTCT | forward primer for BD-CRP |
| BD-CRP-R | TCGACGGATCCCCGGGAATTCCTCCACACGAGTCTTCTTCGG | reverse primer for BD-CRP |
| nLUC-CRP-F | GGAGAGAACACGGGGGACGAGCTCATGACTACTGGTACTCAT | forward primer for nLUC-CRP |
| nLUC-CRP-R | CCTTGTAGTCCATTTGTTGGATCCCCTCCACACGAGTCTTCTT | reverse primer for nLUC-CRP |
| AD-NbSRK-F | GTACCAGATTACGCTCATATGATGGATAAATACGAGCTTGT | forward primer for AD-NbSRK |
| AD-NbSRK-R | ATGCCCACCCGGGTGGAATTCGGTGAGACGAACTTCTCCGCT | reverse primer for AD-NbSRK |
| AD-TaSAPK7-F | GTACCAGATTACGCTCATATGATGGAGAGGTACGAGCTG | forward primer for AD-TaSAPK7 |
| AD-TaSAPK7-R | ATGCCCACCCGGGTGGAATTCGCTGATGTGGAACTCACC | reverse primer for AD-TaSAPK7 |
| AD-TaUBA2C-F | GTACCAGATTACGCTCATATGATGGATCCCTTCTCGAAG | forward primer for AD-TaUBA2C |
| AD-TaUBA2C-R | ATGCCCACCCGGGTGGAATTCGAAATAAGGTGGCACATT | reverse primer for AD-TaUBA2C |
| attB1TaSAPK7 | GGGGACAAGTTTGTACAAAAAAGCAGGCTGCATGGAGAGGTACGAGCTG | forward primer for Donr-TaSAPK7 by gateway |
| attB2TaSAPK7 | GGGGACCACTTTGTACAAGAAAGCTGGGTCGCTGATGTGGAACTCACC | reverse primer for Donr-TaSAPK7 by gateway |
| attB1NbSRK | GGGGACAAGTTTGTACAAAAAAGCAGGCTGCATGGATAAATACGAGCTTGTG | forward primer for Donr-NbSRK by gateway |
| attB2NbSRK | GGGGACCACTTTGTACAAGAAAGCTGGGTCGGTGAGACGAACTTCTCCGCT | reverse primer for Donr-NbSRK by gateway |
| cLuc-TaSAPK7-F | GACGAGCTCGGTACCATGGAGAGGTACGAGCTG | forward primer for cLuc-TaSAPK7 |
| cLuc-TaSAPK7-R | CGAGATCTGGTCGACGCTGATGTGGAACTCACC | reverse primer for cLuc-TaSAPK7 |
| cLUC-NbSRK-F | GCGGAGGTCAGATCTCGTACGCGTATGGATAAATACGAGCTTGTG | forward primer for cLUC-NbSRK |
| cLUC-NbSRK-R | CCTTGTAGTCCATTTGTTGGATCCGGTGAGACGAACTTCTCCGCT | reverse primer for cLUC-NbSRK |
| pGEX-TaSAPK7-F | CTGGTTCCGCGTGGATCCATGGAGAGGTACGAGCTG | forward primer for pGEX-TaSAPK7 |
| pGEX-TaSAPK7-R | GGCCGCTCGAGTCGACCCGGGGCTGATGTGGAACTCACC | reverse primer for pGEX-TaSAPK7 |
| pGEX-TaUBA2C-F | CTGGTTCCGCGTGGATCCATGGATCTAACGAAGAAA | forward primer for pGEX-TaUBA2C |
| pGEX-TaUBA2C-R | GGCCGCTCGAGTCGACCCGGGGTAGTATGGGGGCATGCC | reverse primer for pGEX-TaUBA2C |
| attB1TaUBA2C^∆NLS^ | GGGGACAAGTTTGTACAAAAAAGCAGGC TGCATGAACGGCGCGGTCGCC | forward primer for Donr-TaUBA2C^∆NLS^ |
| attB2TaUBA2C^∆NLS^ | GGGGACCACTTTGTACAAGAAAGCTGGGTC GAAATAAGGTGGCACATT | reverse primer for Donr-TaUBA2C^∆NLS^ |
| qPCRTaUBA2C-F | GTTCGGTGCATTTGGTGGTC | primer used for qRT-PCR |
| qPCRTaUBA2C-R | CGGTATAGTGACGAAGGTCCC |  |
| NbUBC-F | TGGAGGTACATTTAAGCTGACAC | primer used for qRT-PCR |
| NbUBC-R | TCACAGAGCAAAGACTGGATTG |  |
| NbActin-F | AGGCTGTTCTTTCCCTCTATGC | primer used for qRT-PCR |
| NbActin-R | CAACTTCTCCTTCACATCCCTAAC |  |
| W-CDC-F | CAAATACGCCATCAGGGAGAACATC | primer used for qRT-PCR |
| W-CDC-R | CGCTGCCGAAACCACGAGAC |  |
| qPCRCWMVCP-F | TGCAGATTCGCGGTCTGAAT | primer used for qRT-PCR |
| qPCRCWMVCP-R | TCCACACGAGTCTTCTTCGG |  |
| qPCRNbSRK-F | CTGGTTGGGGCATACCCTTT | primer used for qRT-PCR |
| qPCRNbSRK-R | ATCCTCCTTGCTGGATTGGC |  |
| qPCRTaNPR1-F | GGCTTCCCTCGGAAGAGATG | primer used for qRT-PCR |
| qPCRTaNPR1-R | TCTCCTAGTTCGACCTGCCA |  |
| qPCRTaPR1-F | GAGAATGCAGACGCCCAAGC | primer used for qRT-PCR |
| qPCRTaPR1-R | CTGGAGCTTGCAGTCGTTGATC |  |
| qPCRTaRBOHD-F | CAACTGGAAACGCTTGTGGG | primer used for qRT-PCR |
| qPCRTaRBOHD-R | TTCCGCAGCCAAGTAATGGT |  |
