# Supplementary material for: Phosphorylated viral protein evades plant immunity through interfering the function of RNA-binding protein
Source: PLoS Pathog. 2022 Mar 16;18(3):e1010412. doi: 10.1371/journal.ppat.1010412 (PMC8959173; doi:10.1371/journal.ppat.1010412)
Supplement: Attachment — Submitted filename: Response to reviewer.docx [file ppat.1010412.s014.docx]

Responses to Reviewers:

Reviewer #1:

1. The authors did not indicate that SAPK7 is a member of the SnRK2 subfamily of plant protein kinase, substantially understating the potential novelty of their findings.

**Response:** Thanks for your advice. According to your suggestion, we conducted a phylogenetic analysis and showed that SAPK7 is a member of the SnRK2 subfamily of plant protein kinase. Details are showed in Figure S2B and marked in yellow in the manuscript (Line 205-206). In addition, we also discussed the potential novelty of our findings, relevant information is shown in the “Discussion section” and marked in yellow in this article (Line 475-478).

2. Six out of the 15 clones obtained from the Y2H screening with the viral CRP as bait were from the SAPK7 gene (Line 191) but the identity of the rest of the clones need to be shown to indicate if SAPK7 was the only kinase isolated.

**Response:** Thank you for your suggestion. Based on your suggestion, we have provided relevant information in the corresponding location of the manuscript. The total of 15 positive clones were shown in Table S2, and SAPK7 was the only kinase isolated. Details are showed as Line197-198 and marked in yellow.

3. It is also necessary to show the complete phospho-peptide sequences and their phosphorylation scores from the mass spectrum assay (Fig 1b) to support author’s rationale behind their selection of Ser-162 and Ser-165 for further study (Line 141).

**Response:** Thanks for your advice. According to your suggestion, we show the complete phospho-peptide sequences and mass-to-charge ratio from the mass spectrum assay in Table S1. Through 9 independent LC-MS/MS, a total of 8 potential phosphorylation sites were obtained. Considering, S162 and S165 were identified in 6 independent assays (S1 Table), these two sites were chosen for further assays in our study. Detail are showed as Line138-141 and marked in yellow.

4. Fig 3f shows that the CRP S162A/S165A double mutant was not phosphorylated while the S162D/S165D double phosphomimic mutant was highly phosphorylated, even high than the wildtype protein. It suggests that either some sites other than Ser-162 and Ser-165 could be phosphorylated by SAPK7 and their phosphorylation is dependent on the phosphorylation of Ser-162 and/or Ser-165 or their Asp substitutes, or the Ser-to-Asp mutations resulted in new phosphorylation sites for SPAK7. The additional phospho-sites need to be addressed by, for example, mass-spec analysis of the S162D/S165D mutants.

**Response:** Thank you for your suggestion. Following your advice, we analyzed the phosphorylation status of CRP^S162/165D^ by LC-MS/MS. Two phosphorylation sites, S156 and T159, are obtained, and our results suggested that phosphorylation of CRP at S156 and T159 does not affect the pathogenicity of CWMV. Details are shown in Figure S3 and line 222-240 and line 477-483.

5. However, most importantly, the much stronger phosphorylation of the phosphomimic CRP than the phosphorylation of the wildtype CRP (Fig 3f) indicates that the Asp residues at the 162 and/or 165 positions could have different effects on the phosphorylation status of CRP from the effects of the natural phospho-Ser. It suggests that the functional effects of the CRP S162D/S165D mutants observed by the authors in the rest of the manuscript probably have limited biologically significance.

**Response:** Thanks for your advice. According to your suggestion, we analyzed the phosphorylation status of CRP^S162/165D^ by LC-MS/MS. We identified two secondary phosphorylation sites, S156 and T159, and the functions of these two sites were studied. The results showed that phosphorylation of CRP at S156 and T159 does not affect the pathogenicity of CWMV. Details are shown in Figure S3 and line 222-240 of “Results section” and line 477-483 of “Discussion section”.

6. The authors expressed TaUBA2C proteins with either of its RNA binding domains deleted for assessing their importance in forming nucleic speckles (Line 365, Fig 8a), in vitro DNA and RNA binding (Line 398, Fig 8e) and cell death (Line 406, Fig 8f). Removing a large segment from a protein may substantially disrupt its 3-D structure necessary for sustaining its biochemical functions. If this issue cannot be addressed, these data should be removed.

**Response:** Thank you for your suggestion. Following your advice, we have removed this part of the data from the revised manuscript.

7. The authors used RNA-immunoprecipitation (RIP) assays to show that the phosphomimic viral CRP could interfere with the binding of TaUBA2C to the pre-mRNAs of the TaNPR1, TaPR1, and TaRBOHD genes and downregulate their expression. In Fig 7d, assays of wildtype plants infected with wildtype virus were missing making the plants infected with the mutant viruses uncontrolled. The RIP assays presented in Fig 8g lack controls for the three target genes. Also, the wildtype plants that do not express any His-tagged proteins were not adequate controls for the UBA2C-His bait protein because amounts of non-specific proteins bound to the affinity resin were likely much less than the bound UBA2C-His.

**Response:** Thanks for your advice. This was a mistake during our experiment, and based on your suggestion, we've did the experiment again. Details are shown in Figure 7H and Figure 8E, line 372-384 and line 430-443.

8. In Fig 7 or Fig 8, the authors did not show the expression levels of the 3 target genes in the UBA2C-overexpressing transgenic plants infected with the wildtype or mutant viruses, so unable to address the possibility that high levels of UBA2C may overcome the effect of the mutant CRPs.

**Response:** Thank you for your suggestion. Following your advice, we have added the analysis of the expression levels of three target genes in wildtype or mutant virus-infected UBA2C overexpression transgenic plants in Figure 8G, and details are shown in line 450-459.

9. This is also important for providing measurements of total input of the pre-mRNAs of the 3 target genes in the RIP assays presented in Fig 8h. Ideally, similar levels of pre-mRNA input for a particular gene among plants infected with different viruses would make the assay more reliable.

**Response:** Thanks for your advice. According to your suggestion, we added relevant data to the materials and methods section, details are shown in line 749-769.

10. The results of assaying peroxide production (Fig 7b) do not demonstrate clear-cut differences between the samples. Are there any quantitative assay methods that can be used here?

**Response:** Thank you for your suggestion, following your advice, the relative DAB staining intensity was calculated using IMAGEJ software (http://rsbweb.nih.gov/ij). The relevant results are shown in Figure 7B, 7D, 7F.

11. Fig 1b was described in the figure legend as CBB stained gel. Why have the CBB-stained protein bands turned black while the marker bands remained blue?

**Response:** Thanks for your advice. This is an error in our description, Figure 1B (upper panel) is a western blot analysis to detect CRP-GFP purified from the inoculated protoplasts. We have corrected it, details are shown in line 1010-1012.

12. Some details in methods are missing, for example, 1) For the RNA- and DNA-binding activity assay described on P. 694, no input quantities of proteins or nucleic acids are shown. 2) How the plasmids containing the TaUBA2C gene with deletions of the RNA biding domains were made are not described. 3) Primers for qPCR detection of the TaNPR1, TaPR1, and TaRBOHD genes in both RT-PCR and RIP experiments cannot be found in the Supplemental Table.

**Response:** Thank you for your suggestion, we have supplemented the relevant data according to your advice. 1) Approximately 100 ng of recombinant proteins was incubated with the resins in 400 μL KHN buffer, shown in line 742. 2) Reviewer suggested that removing a large segment from a protein may substantially disrupt its 3-D structure necessary for sustaining its biochemical functions. So, data related to the TaUBA2C deletion mutant has been removed. 3) Primers for qPCR detection of the TaNPR1, TaPR1 and TaRBOHD genes in both RT-PCR and RIP experiments have been added to the Supplemental Table 3.

Reviewer #2:

1. The authors used mass spectra for identification of eight potential phosphorylated sites on CRP, including both serine and threonine residues. However, only two serine residues were used for further analyses. More importantly, after mass spectra identification, no in vivo experiment was performed to provide evidence that the chosen S162/S165 are the predominant phosphorylation sites. Thus, at least the phosphorylation levels of the WT and S162/165A versions of CRP need to be compared in plant cells.

**Response:** Thank you for your suggestion, following your advice, the phosphorylation levels of the WT and S162/165A versions of CRP had been compared in Figure 1C, and a description is added in the corresponding position of the manuscript (Line 141-149).

2. For most of interaction identification and verification, multiple controls are missing. For instance, in Figure 3a, only the AD-TaSAPRK7 and BD-CRP pair is shown, but the controls for exclusion of auto-activation are not presented, including the pair of AD-TaSAPRK7 and empty BD, as well as the pair of empty AD and BD-CRP. Similarly, in the BiFC and LCI assays in Figure 3b and 3c, only the pair of TaSAPRK7 and CRP is showed, but their combination with empty vectors are not included. Similar problems can be found in Figure 4 and Figure 5. Because false positive signals often can be detected in these interaction assays, it is important to verify the interaction and include all controls in the manuscript.

**Response:** Thanks for your advice. This was a mistake during our experiment, and based on your suggestion, we have added the missing controls in Figure 3, Figure 4 and Figure 5.

3. In Figure 5c, an in vitro pull-down assay was used to confirm the effect of phosphorylation on the interaction between CRP-his and GST-TaUBA2C. The authors may use the proteins expressed in bacteria for in vitro pull-down, and found the S162/S165 version of CRP did not interact with GST-TaUBA2C. However, because CRP phosphorylation does not happen in bacteria, the effect of this mutant may be from protein structure changes. Thus, the authors should consider carefully whether the effect of potential structure changes of the mutant used throughout the manuscript.

**Response:** Thank you for your suggestion. Based on your advice, we have reviewed the relevant reference, as previously reported (Boris Macek et al. Molecular & Cellular Proteomics 7:299–307, 2008.), phosphorylation can also occur in bacteria. Moreover, Zhang et al. (New Phytol, 2018) and Gao et al. (Plant Cell, 2020) have also used this method.

4. 1) The authors used Ala and Asp to replace the serine residues, resulting in loss and mimic of phosphorylation on CRP. In line 149, the authors claimed that Asp is a phosphorylatable residue. In my opinion, the mimic function of Asp may be contributed from introduction of a negative charge, similar to phosphorylation. The authors may clarify it or cite reference to support their statement.

**Response:** Thanks for your advice. This is an error in our description, we have corrected it, details are shown in line 155-157. In addition, we have cited reference (Zhang et al. New Phytol, 2018 and Gao et al. Plant Cell, 2020) to support our statement, details are shown in line 141.

5. It is better to quantify the Trypan blue and DAB staining data in Figure 7 and 8.

**Response:** Thank you for your suggestion, following your advice, the relative Trypan blue or DAB staining intensity was calculated using IMAGEJ software (http://rsbweb.nih.gov/ij). The relevant results are shown in Figure 7. Other reviewer suggested that removing a large segment from a protein may substantially disrupt its 3-D structure necessary for sustaining its biochemical functions. So, data related to the TaUBA2C deletion mutant has been removed. The Trypan blue and DAB staining data in Figure 8 had been removed.

6. The sample sizes of all symptom analysis should be included in figure legends.

**Response:** Thank you for your advice. Based on your suggestion, the sample sizes of all symptom analysis had been added in figure legends.

7. There are some spelling mistakes and English writing needs to be improved throughout the manuscript.

**Response:** Thanks for your suggestion, we carefully checked the spelling of words in the manuscript and have made revisions.


Reviewer #3:

1. To evaluate the thoroughness of the study, it is important to know how many test plants per CWMV version were inoculated in the three independent trials, what criteria were used to classify the expression of symptoms, and how variable the severity of symptom was within a group of test plants infected with a virus variant. What were the Infection rates, and was symptom scoring performed only after 21 days? Were differences in infectivity and symptom development found among the different host plants for a particular virus mutant? The data should be presented as a supplementary material, e.g. in a summary table, and information should be added at respective positions in the manuscript e.g. material and methods.

**Response:** Thank you for your suggestion. Following your advice, we have added relevant information in the Materials and Methods section. Details are shown in line 583-588 and Fig S10.

2. Inconsistency 111 “non-phosphorylatable S162 and S165 (e.g., CWMVS162/165A), the inoculated *N. benthamiana* and wheat plants developed milder disease symptoms” vs. 151 “CWMVS162A - or CWMVS165A 151 -inoculated plants showed mild mosaic symptoms, similar to that on the WT CWMV-inoculated plants” Clear statement whether double or single mutations are meant.

**Response:** Thanks for your advice. This is an error in our description, we have corrected it following your suggestion, details are shown in line 105-109 and line 155-157.

3. The materials and methods section should contain all the necessary information and/or references to understand how the experiments were performed.  For example, there is often a lack of information about the manufacturers of the materials used, e.g. for antibodies, and the dilutions used for Western blot analyses or the method applied in yeast two-hybrid assays. What are the positive and negative controls applied for BiFC and LCI assay?

**Response:** Thank you for your suggestion. Following your advice, we have added relevant information in the Materials and Methods section. Details about the positive and negative controls applied for BiFC and LCI assay are shown in line 647-648 and line 660-661.

4. Figure S1: Image of leaves implies that after 6 dpi is in the empty vector control GFP expression recovered which is not detectable in the western blot analysis below.

**Response:** Thanks for your advice. According to your suggestion, we repeated the experiment 6 times, by 6 dpi, the green fluorescence in the 16c plant leaves expressing sGFP+EV was silenced (S1A Fig). Consistent with this result, no GFP protein was detected in these GFP-silenced 16c plant leaves through western blot analysis (S1B Fig).

5. Please indicate if the clones representing 355 aa of SAPK7 are truncated or full-length.

**Response:** Thank you for your suggestion. This polypeptide represents the full-length of TaSAPK7. Details are shown in line 201-202.

6. CWMV encoding a double non-phosphorylation CRP mutant lead in plants to milder symptoms as a WT virus, but the mutant version does not interact with UBA2C to suppress cell death and H2O2 production. If UBA2C- CRP interaction leading to suppression of cell death and H2O2 production would be the main determinant of CWMV pathogenicity, the non-phosphorylation form of CRP would lead to symptomless plants or probably loss of infection. This CRP version should be unable to enter the nucleus and to interact with UBA2C. The data shows infectivity of CWMV encoding AA version of CRP.

**Response:** Thanks for your advice. This is an error in our description, in this study, the CWMV^S162/165A^-inoculated plants did not show clear mosaic symptoms (Fig 2A). We have corrected this error, details are shown in line 162-163.

7. This issue should be discussed Why is the double D mutant of CRP phosphorylated, but not the double A mutant in in vitro phosphorylation assays? D and A can both not be phosphorylated by a kinase. D mimics only the charge of a phosphorylated aa and is thus regraded as a phosphorylation (charge)-mimicking mutant. At various points in the manuscript, this is not neatly distinguished and discussed

**Response:** Thank you for your suggestion. Following your advice, we analyzed the phosphorylation status of CRP^S162/165D^ by LC-MS/MS. We identified two secondary phosphorylation sites, S156 and T159, so, the double D mutant of CRP was phosphorylated. The results showed that phosphorylation of CRP at S156 and T159 does not affect the pathogenicity of CWMV. Details are shown in Figure S3 and line 222-240 of Results section and line 474-483 of Discussion section.

8. 51 Protein

**Response:** Thanks for your advice. We have corrected the spelling mistakes in line 49.

9. 68 by dead cells?

**Response:** Thanks for your advice. We have corrected this mistakes in line 66.

10. 70 Regulation of gene expression by post-transcriptional modification in the context of defense is important for PCD and plant immunity

**Response:** Thanks for your advice. We have corrected it in line 68-69.

11. 107 remove to show

**Response:** Thanks for your advice. We have removed “to show” in line 104.

12. 108 Using a CWMV mutant encoding a CRP no longer phosphorylatable at S162 and S165 (e.g. CWMVS162/165A 109 ), inoculated N. benthamiana and wheat plants developed milder disease symptoms and contained much less virus compared with CWMV wild-type (WT) infection. Meaning unclear „mainly due to cell death and H2O2 production “better split in two sentence and clarify meaning.

**Response:** Thanks for your advice. Following your suggestion, we have revised this description, details are shown in line 105-109.

13. 131 PEG4000-based method

**Response:** Thanks for your advice. We have revised this description according to your suggestion, details are shown in line 128-129.

14. 132 After overnight incubation at room temperature…and CRP-GFP enriched by immunoprecipitation. Comparative western blot analysis using GFP and phosphoserine-specific antibodies revealed in protoplasts expressing the fusion protein a 45 kDa band indicative for phosphorylated CRP-GFP.  Include that GFP expressing samples were processed in parallel as a control

**Response:** Thanks for your advice. Following your suggestion, we have revised this description, details are shown in line 129-134.

15. 137 To identify the phosphorylation sites in CRP, the GFP-tagged protein was purified by affinity chromatography from total protein extracts of expressing wheat protoplast.

**Response:** Thanks for your advice. We have revised this description according to your suggestion, details are shown in line 134-136.

16. 148 amino acid position 162 and/or 165.

**Response:** Thanks for your advice. Based on your suggestion, we have made changes, details are shown in line 157.

17. 149 a mutant mimicking negative charge introduced by phosphorylation, negative-charge mimicking mutant.

**Response:** Thanks for your advice. Based on your suggestion, we have made changes, details are shown in line 158-159.
