# Supplementary material for: Phosphorylated viral protein evades plant immunity through interfering the function of RNA-binding protein
Source: PLoS Pathog. 2022 Mar 16;18(3):e1010412. doi: 10.1371/journal.ppat.1010412 (PMC8959173; doi:10.1371/journal.ppat.1010412)
Supplement: Attachment — Submitted filename: Response to reviewers.docx [file ppat.1010412.s015.docx]

Responses to Reviewers:

Reviewer #2:

1. 55 Explain BSMV abbreviation

**Response:** Thank you for your suggestion, following your advice, we explained BSMV abbreviation in Line 55 and marked in yellow in the manuscript.

2. 57 For geminiviruses, there are extensive review articles on PTMS that regulate the functions of viral proteins, including SnRK1, possibly cite them here (W. Shen and L. Hanley-Bowdoin Curr Opin Virol 2021 Vol. 47 Pages 18-24; R. M. Teixeira, M. A. Ferreira, G. A. S. Raimundo and E. P. B. Fontes Microorganisms 2021 Vol. 9 Issue 4; L. Hanley-Bowdoin, E. R. Bejarano, D. Robertson and S. Mansoor Nat Rev Microbiol 2013 Vol. 11 Issue 11 Pages 777-88)

**Response:** Thanks for your advice. According to your suggestion, we cited these references in the manuscript. Details are showed in Line 57 and Line 478-479 and marked in yellow.

3. 134 remove “include that”?

**Response:** Thanks for your suggestion. We have removed “include that” in Line 134.

4. 151 at this point of the manuscript the conclusion is to early, better “suggest that S162 and S165 phosphorylation sites are important”

**Response:** Thank you for your advice. We have corrected it following your suggestion, details are shown in Line 151 and marked in yellow.

5. 196 remove “that”

**Response:** Thanks for your suggestion. We have removed “that” in Line 196.
